# Evaluation of Stability of Amylose Inclusion Complexes Depending on Guest Polymers and Their Application to Supramolecular Polymeric Materials

**DOI:** 10.3390/biom7010028

**Published:** 2017-03-15

**Authors:** Tomonari Tanaka, Atsushi Tsutsui, Kazuya Tanaka, Kazuya Yamamoto, Jun-ichi Kadokawa

**Affiliations:** 1 Department of Biobased Materials Science, Graduate School of Science and Technology, Kyoto Institute of Technology, Matsugasaki, Sakyo-ku, Kyoto 606-8585, Japan; atsushi.tsutsui@gmail.com; 2 Department of Chemistry, Biotechnology, and Chemical Engineering, Graduate School of Science and Engineering, Kagoshima University, 1-21-40 Korimoto, Kagoshima 860-0065, Japan; k1987386@kadai.jp (K.T.); yamamoto@eng.kagoshima-u.ac.jp (K.Y.)

**Keywords:** amylose, dissociation, film, inclusion complex, supramolecular polymer, vine-twining polymerization

## Abstract

This paper describes the evaluation of the stability of amylose–polymer inclusion complexes under solution state in dimethyl sulfoxide (DMSO) depending on guest polymers. The three complexes were prepared by the vine-twining polymerization method using polytetrahydrofuran (PTHF), poly(ε-caprolactone) (PCL), and poly(l-lactide) (PLLA) as guest polymers. The stability investigation was conducted at desired temperatures (25, 30, 40, 60 °C) in DMSO solutions of the complexes. Consequently, the amylose–PTHF inclusion complex was dissociated at 25 °C, while the other complexes were stable under the same conditions. When the temperatures were elevated, the amylose–PCL and amylose–PLLA complexes were dissociated at 40 and 60 °C, respectively. We also found that amylose inclusion supramolecular polymers which were prepared by the vine-twining polymerization using primer-guest conjugates formed films by the acetylation of amylose segments. The film from acetylated amylose–PLLA supramolecular polymer had higher storage modulus than that from acetylated amylose–PTHF supramolecular polymer, as a function of temperature.

## 1. Introduction

Amylose is an abundant natural polysaccharide as an energy resource present in starch [1]. Besides this vital role in nature, it is a well-known functional polymer and acts as a host molecule to form supramolecular inclusion complexes with various hydrophobic guest molecules, owing to its left-handed helical conformation and hydrophobic nature inside the cavity [2]. However, a limitation of binding directly toward polymeric guest molecules into the cavity has been found, because the driving force for complexation is implied by weak hydrophobic interaction [3,4,5,6,7,8,9,10,11,12]. We have developed an efficient method for the formation of inclusion complexes from amylose and polymeric guest molecules by means of enzymatic polymerization [13,14,15,16,17,18,19,20].

**Figure 1:**
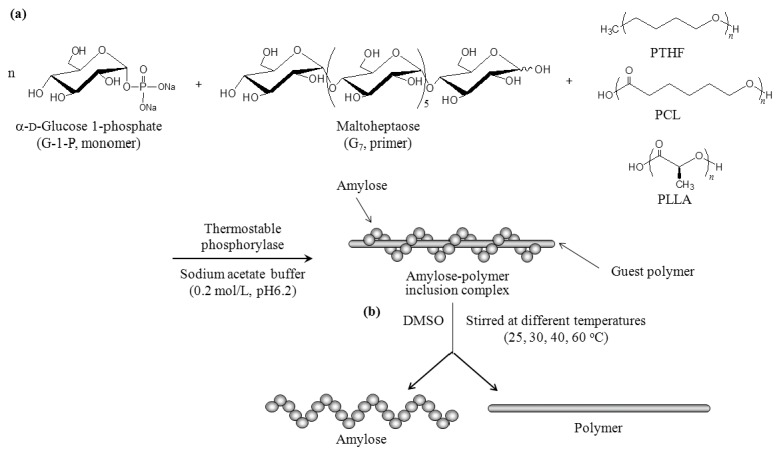
Preparation of: (**a**) inclusion complexes by vine-twining polymerization using polytetrahydrofuran (PTHF), poly(ε-caprolactone) (PCL), and poly(l-lactide) (PLLA) as guest polymers and (**b**) their dissociation under solution state in dimethyl sulfoxide (DMSO).

Amylose with well-defined structure is synthesized by enzymatic polymerization catalyzed by phosphorylase [21,22,23,24], as the enzymatic approach has been well accepted as a powerful tool to obtain polysaccharides with precisely controlled structure [16,20,25]. The phosphorylase-catalyzed enzymatic polymerization occurs by using *α*-d-glucose 1-phoshate (G-1-P) as a monomer and maltooligosaccharide as a primer according to the following reversible reaction: (*α*(1→4)-G)*_n_* + G-1-P ⇄ (*α*(1→4)-G)*_n+1_* + P. In the reaction, a glucose (G) residue is transferred from G-1-P to the non-reducing 4-OH propagating terminus of a *α*(1→4)-glucan chain, liberating inorganic phosphate (P). We have found that inclusion complexes are gradually formed with the progress of propagation when the phosphorylase-catalyzed enzymatic polymerization was carried out in the presence of hydrophobic guest polymers dispersed in an aqueous buffer solvent (Figure 1a) [13,14,15,16,17,18,19,20]. The process of propagation from the shorter *α*(1→4)-glucan chain (maltooligosaccharide primer) to the longer *α*(1→4)-glucan chain (amylose) efficiently induces complexation with polymeric guest molecules. As the process of such complexation is similar to the way that vines of plants grow twining around a rod, the authors have named this polymerization approach “vine-twining polymerization”. In previous studies, hydrophobic polyethers such as polytetrahydrofuran (PTHF) [26,27] and hydrophobic polyesters such as poly(ε-caprolactone) (PCL) and poly(l-lactide) (PLLA) [28,29,30] have been found to form inclusion complexes with amylose by the vine-twining polymerization (Figure 1a).

**Figure 2:**
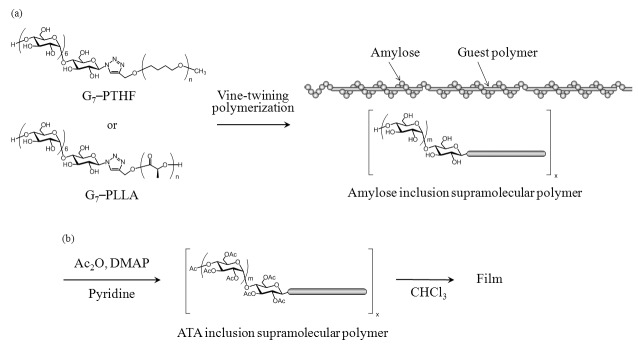
Preparation of: (**a**) amylose triacetate (ATA) inclusion supramolecular polymers and (**b**) their films. DMAP: *N,N*-dimethyl-4-aminopyridine.

By means of the vine-twining polymerization method, we have achieved the synthesis of inclusion supramolecular polymers which are composed of a continuum of the amylose–PTHF and amylose–PLLA inclusion complexes [31,32,33,34]. When primer (maltoheptaose, G_7_)-guest conjugate substrates (G_7_–PTHF and G_7_–PLLA) were used in the vine-twining polymerization, where the termini of the guest polymers were covalently connected with the reducing end of G_7_ via a triazole ring (Figure 2a), a propagating amylose chain which was started from the G_7_ segment in the G_7_-guest conjugate by the phosphorylase catalysis included the guest segment in the other substrate. This special type of the vine-twining polymerization among the substrates successively takes place, giving rise to the inclusion supramolecular polymers composed of amylose and guest polymer PTHF or PLLA.

To understand the natures of amylose–polymer inclusion complexes further, in this study we investigated the evaluation of their stability under solution state in dimethyl sulfoxide (DMSO) depending on guest polymers. On the basis of the findings, we also evaluated the mechanical properties of amylose triacetate (ATA)–PTHF and ATA–PLLA supramolecular polymeric films as a function of temperature, which were found to be obtained by acetylation of the original amylose inclusion supramolecular polymers. In a previous study, we also prepared a supramolecular film via the formation of a hydrogel by the vine-twining polymerization approach using the appropriately designed graft copolymer (i.e., carboxymethyl cellulose-*graft*-PCL) [35]. In this system, the enzymatically produced amyloses formed inclusion complexes with grafted PCL chains in the intermolecular graft copolymers, which acted as cross-linking points, resulting in network structures. Such network structures contributed to forming the hydrogel from the vine-twining polymerization solution, which was easily converted into the supramolecular film by drying. The present study provides a new type of supramolecular film constructed from linear polymeric structures, which are based on a completely different preparative strategy from that of the previous study.

## 2. Results and Discussion

### 2.1. Evaluation of the Stability of Inclusion Complexes Under Solution State

**Figure 3:**
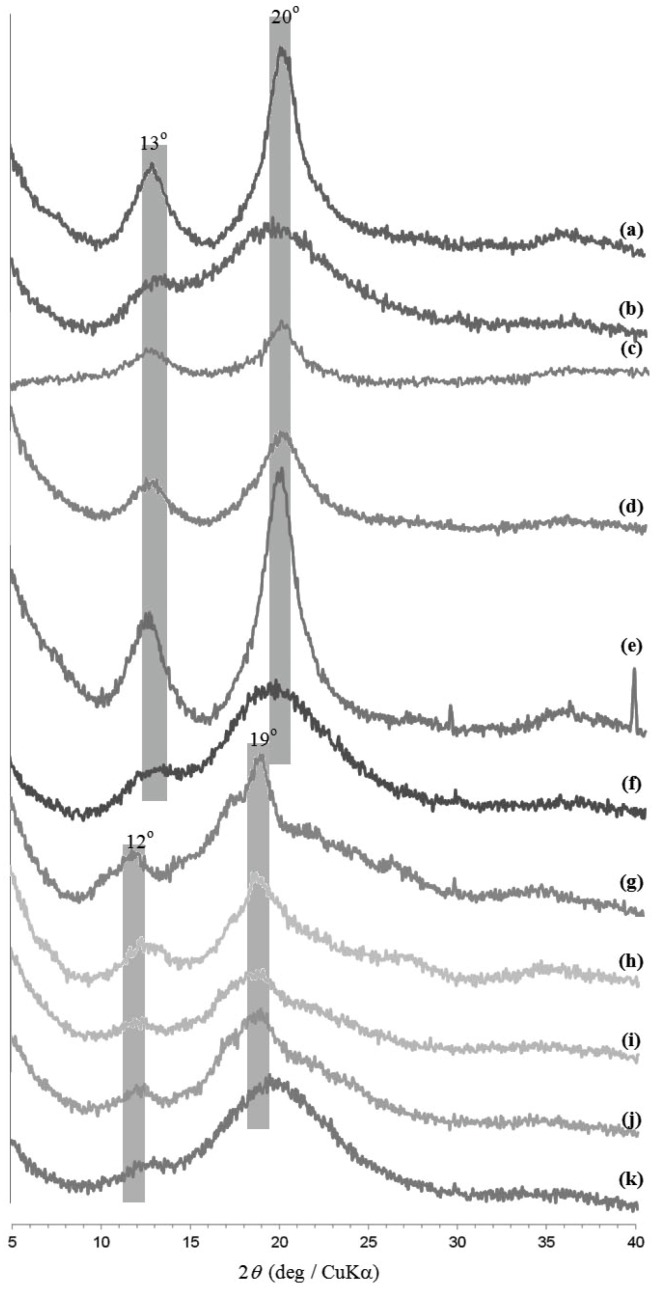
X-ray diffraction (XRD) profiles of: (**a**) amylose–PTHF inclusion complex; (**b**) chloroform-insoluble fraction by its treatment in DMSO at 25 °C; (**c**) amylose–PCL inclusion complex, chloroform-insoluble fractions by its treatment in DMSO at (**d**) 25 °C, (**e**) 30 °C, and (**f**) 40 °C; (**g**) amylose–PLLA inclusion complex, chloroform-insoluble fractions by its treatment in DMSO at (**h**) 25 °C, (**i**) 30 °C, (**j**) 40 °C, and (k) 60 °C.

The amylose–polymer inclusion complexes were prepared by vine-twining polymerization using PTHF (Number-average molecular weight (*M*_n_) = 2800 by ^1^H nuclear magnetic resonance (NMR)), PCL (*M*_n_ = 2400 by ^1^H NMR), and PLLA (*M*_n_ = 2600 by ^1^H NMR) according to literature procedures (Figure 1a) [27,29,30]. The structures of the inclusion complexes were supported by the ^1^H NMR spectra (see Materials and Methods section). The X-ray diffraction (XRD) patterns of the products were identical with those of the literature data; 2*θ* = ca. 13 and 20° for 6_1_-helix from PTHF and PCL, 2*θ* = ca. 12 and 19° for 7_1_-helix from PLLA (Figure 3a,c,g).

**Figure 4:**
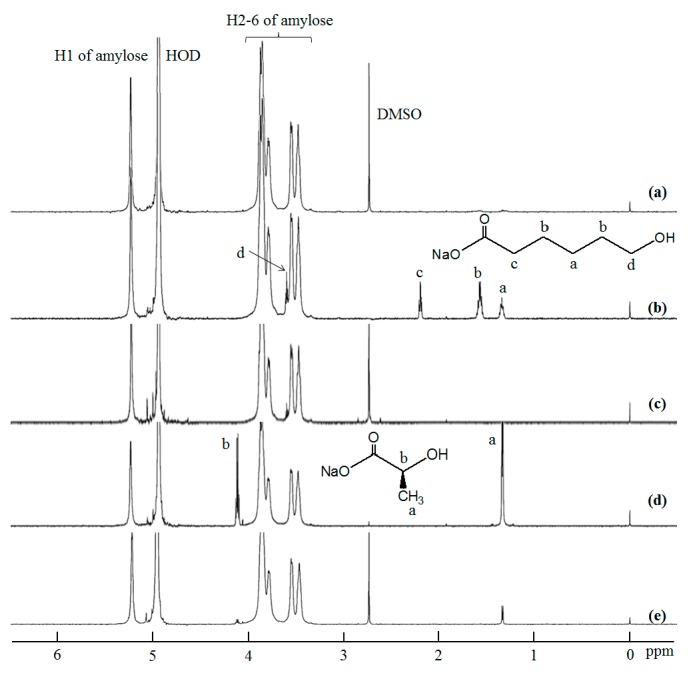
^1^H nuclear magnetic resonance (NMR) spectra of: (**a**) chloroform-insoluble fraction by treatment of amylose–PTHF inclusion complex in DMSO at 25 °C; chloroform-insoluble fractions by treatment of amylose–PCL inclusion complex in DMSO at (**b**) 30 °C and (**c**) 40 °C; and chloroform-insoluble fractions by treatment of amylose–PLLA inclusion complex in DMSO at (**d**) 40 °C and (**e**) 60 °C (0.5 M NaOD/D_2_O).

**Figure 5:**
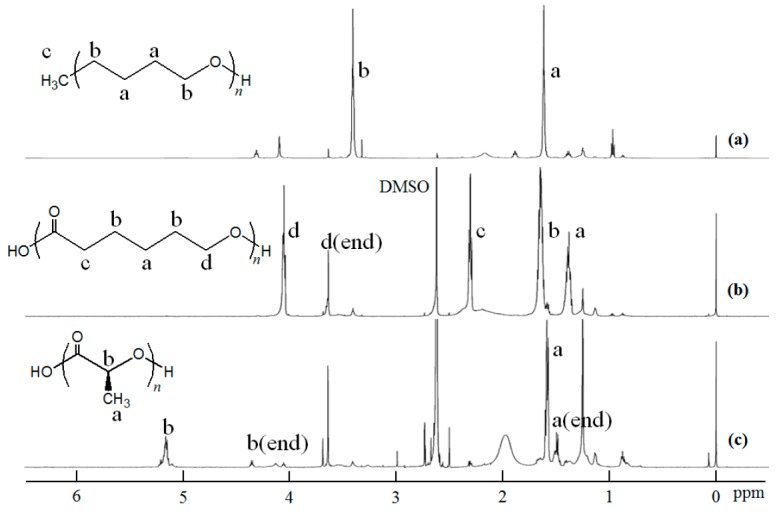
^1^H NMR spectra of: (**a**) chloroform-soluble fraction by treatment of amylose–PTHF inclusion complex in DMSO at 25 °C; (**b**) chloroform-soluble fraction by treatment of amylose–PCL inclusion complex in DMSO at 40 °C; and (**c**) chloroform-soluble fraction by treatment of amylose–PLLA inclusion complex in DMSO at 60 °C (CDCl_3_).

As the inclusion complexes were soluble in DMSO, the stability investigations under solution state were conducted in DMSO. After the inclusion complexes were treated in DMSO for 12 h at desired temperatures, the products were separated into chloroform-soluble and -insoluble fractions (Figure 1b). The XRD pattern of the chloroform-insoluble fraction after the treatment of the amylose–PTHF inclusion complex at 25 °C was broadened, which is completely different from that before treatment (Figure 3a,b). On the other hand, the XRD patterns of the chloroform-insoluble fractions from the other complexes remained intact by treatment at 25 °C (Figure 3c,d,g,h). These results indicate that the dissociation of the amylose–PTHF inclusion complex probably occurred, while the amylose–PCL and –PLLA inclusion complexes were stable at that temperature. Indeed, the ^1^H NMR spectrum of the chloroform-insoluble fraction (0.5 M NaOD/D_2_O) from the amylose–PTHF inclusion complex shows signals assignable to amylose, but does not show signals due to PTHF (Figure 4a). Furthermore, the ^1^H NMR spectrum of the chloroform-soluble fraction (CDCl_3_) from the same complex supports the structure of PTHF (Figure 5a).

By elevating the treatment temperature of the amylose–PCL inclusion complex to 30 °C, the XRD pattern of the chloroform-insoluble fraction was not significantly changed from the original pattern (Figure 3e). Moreover, the ^1^H NMR spectrum of the same sample (0.5 M NaOD/D_2_O) showed signals assignable to both amylose and 6-hydroxycaproic acid sodium salt (the hydrolyzed product from PCL under alkaline condition in 0.5 M NaOD/D_2_O) (Figure 4b), supporting that the inclusion complex retains stability after treatment at that temperature. When the treatment temperature of this inclusion complex was elevated to 40 °C, the XRD pattern of the chloroform-insoluble fraction was broadened (Figure 3f) as same as that treated from the amylose–PTHF inclusion complex at 25 °C. The ^1^H NMR spectrum of this fraction (0.5 M NaOD/D_2_O) only showed a signal assignable to amylose (Figure 4c), whereas the ^1^H NMR spectrum of the chloroform-soluble fraction (CDCl_3_) supports the structure of PCL (Figure 5b). These results suggest that the amylose–PCL inclusion complex was dissociated by treatment in DMSO at 40 °C.

When the treatment temperatures of the amylose–PLLA inclusion complex were elevated up to 40 °C, the XRD patterns of the chloroform-insoluble fractions were not significantly changed from the original data (Figure 3h–j). Furthermore, in the ^1^H NMR spectrum of the chloroform-insoluble fraction by the treatment at 40 °C in 0.5 M NaOD/D_2_O (Figure 4d), the signals due to both amylose and l-lactic acid sodium salt (the hydrolyzed product from PLLA under alkaline condition in 0.5 M NaOD/D_2_O) are detected. By further elevating the treatment temperature of the amylose–PLLA inclusion complex to 60 °C, the XRD pattern of the chloroform-insoluble fraction is broadened, which is completely different from that before treatment (Figure 3h,k). Indeed, the signals assignable to amylose are only detected in the ^1^H NMR spectrum of this fraction (0.5 M NaOD/D_2_O) (Figure 4e). Furthermore, the ^1^H NMR spectrum of the chloroform-soluble fraction (CDCl_3_) supports the structure of PLLA (Figure 5c). These results indicate the occurrence of the dissociation of the amylose–PLLA inclusion complex by treatment in DMSO at 60 °C.

Based on the above results, we conclude the following order for the stability of the inclusion complexes under solution state in DMSO: amylose–PLLA inclusion complex > amylose–PCL inclusion complex > amylose–PTHF inclusion complex. The difference in stability of the complexes is probably owing to the bulkiness of the guest polymers. The slenderest structure of PTHF induces the easier dissociation of the complex. With increasing the bulkiness by incorporating carbonyl groups into the PCL and PLLA main-chains and further methyl group into the PLLA main-chain, the guest polymers slip out from cavity of amylose less easily, leading to an enhancement of the stability of the complex in DMSO.

### 2.2. Preparation and Mechanical Properties of ATA Supramolecular Polymeric Films

**Figure 6:**
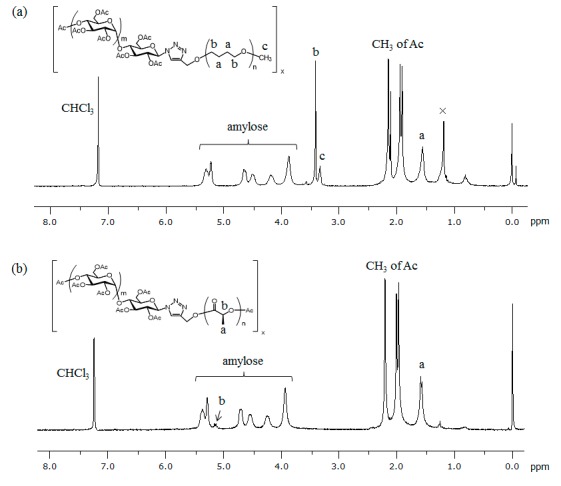
^1^H NMR spectra of: (**a**) ATA–PTHF and (**b**) ATA–PLLA supramolecular polymers (CDCl_3_).

**Figure 7:**
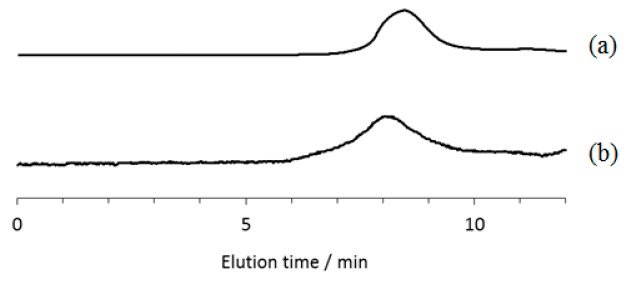
Gel permeation chromatography (GPC) traces of (**a**) ATA–PTHF and (**b**) ATA–PLLA supramolecular polymers.

Based on the above results, we also investigated the evaluation of the mechanical properties of ATA–PTHF and –PLLA supramolecular polymeric materials as a function of temperature. We found that the supramolecular polymeric films were obtained by acetylation of amylose segments, which were suitably subjected to dynamic mechanical analysis (DMA) measurement for the evaluation. The ATA supramolecular polymers were prepared from amylose–PTHF and –PLLA inclusion supramolecular polymers which were synthesized by the vine-twining polymerization using G_7_–PTHF and G_7_–PLLA, respectively [31,33], by the acetylation reaction using acetic anhydride (Ac_2_O) in pyridine in the presence of *N,N*-dimethyl-4-aminopyridine (DMAP) (Figure 2b). The ^1^H NMR spectra of the acetylated products showed signals assignable to the methyl of introduced acetyl groups, the acetylated amylose, and the guest polymer segments (Figure 6). The degrees of substitution on hydroxy groups by acetyl groups were determined by the ^1^H NMR spectra to be quantitative in both the supramolecular polymers. Figure 7 shows the gel permeation chromatography (GPC) traces of acetylated products, which show peaks with unimodal profiles. The *M*_n_ values of ATA–PTHF and –PLLA supramolecular products were calculated to be 1.3 × 10^5^ g·mol^−1^ (Weight-average molecular weight (*M*_w_)/*M*_n_ = 3.6) and 5.4 × 10^5^ g·mol^−1^ (*M*_w_/*M*_n_ = 5.2), respectively. The *M*_n_ values of primer–guest conjugate substrates (G_7_–PTHF and G_7_–PLLA) used for the vine-twining polymerization were much smaller (3.3 × 10^3^ g·mol^−1^ and 2.8 × 10^3^ g·mol^−1^, respectively), suggesting that the inclusion complexation by amylose in the supramolecular polymers was not dissociated under the acetylation condition.

**Figure 8:**
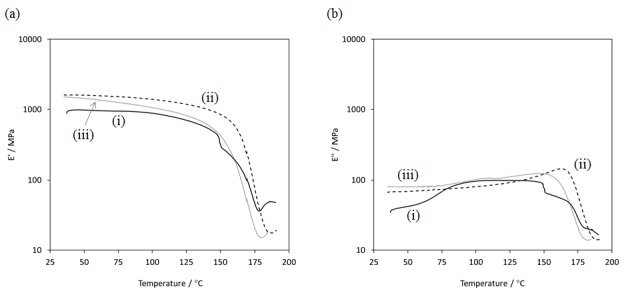
Dynamic mechanical analysis of (i) ATA–PTHF and (ii) ATA–PLLA supramolecular polymeric films, and (iii) potato ATA. (**a**) storage modulus (E′); (**b**) loss modulus (E′′).

We found that the present ATA inclusion supramolecular polymers were soluble in common organic solvents and could easily form films, although the amylose–polymer supramolecular polymers before acetylation were mostly insoluble in such organic solvents. Accordingly, flexible cast films were obtained from chloroform solutions of the ATA–PTHF and –PLLA supramolecular products. Figure 8 shows the DMA measurements of the resulting films from ATA supramolecular polymers and potato ATA (*M*_n_ = 1.8 × 10^5^ g·mol^−1^, *M*_w_/*M*_n_ = 7.2 by GPC), which was prepared by the same acetylation procedure from a commercial potato amylose sample. Storage moduli (E′) of the ATA–PLLA supramolecular polymeric film were higher than those of the ATA–PTHF supramolecular polymeric film and potato ATA on the whole temperature range. The descent curve of E′ of the ATA–PLLA supramolecular polymeric film shifted to the higher temperature region compared with that of the ATA–PTHF supramolecular polymeric film and potato ATA (Figure 8a), suggesting that PLLA segments included by amylose had enhanced the stability of the supramolecular polymeric film under heating. E′ of the ATA–PTHF supramolecular polymeric film dropped off at 150 °C, suggesting that the inclusion by amylose might be dissociated in the ATA–PTHF supramolecular polymeric film under heating. There is no such behavior in the case of the ATA–PLLA supramolecular polymeric film. Furthermore, loss moduli (E′′) of the ATA–PLLA supramolecular polymeric film are shown to be higher than those of the ATA–PTHF supramolecular polymeric film at 150–165 °C (Figure 8b). These DMA results support that the film from ATA–PLLA supramolecular polymer is more stable at high temperature than that from ATA–PTHF supramolecular polymer and potato ATA.

## 3. Materials and Methods

### 3.1. Materials

The primer G_7_ was prepared by selective cleavage of one glucosidic bond of β-cyclodextrin under acidic conditions [36]. The guest polymers PTHF, PCL, and PLLA were synthesized by ring-opening polymerization of tetrahydrofuran, ε-caprolactone, and l-lactide monomers initiated with methyl trifluoromethanesulfonate, 6-hydroxycaproic acid, and l-lactic acid, respectively [37,38,39]. Amylose–PTHF and –PLLA inclusion supramolecular polymers were synthesized according to the literature procedures [31,33]. Thermostable phosphorylase from *Aquifex aeolicus* VF5 was supplied from Ezaki Glico Co. Ltd. (Osaka, Japan) [23,40,41]. Potato amylose was purchased from Sigma-Aldrich Co. LLC. (St. Louis, MO, USA). Other reagents and solvents were commercially available and used without further purification.

### 3.2. Preparation of Amylose–Polymer Inclusion Complexes

A typical experimental procedure was as follows. A mixture of the guest polymer (50 mg) with sodium acetate buffer (pH 6.2, 0.2 mol/L, 4.0 mL) was sonicated to obtain a dispersion. After the addition of G_7_ (1.2 mg, 1.0 mmol), G-1-P disodium salt (76.1 mg, 0.25 mmol), and thermostable phosphorylase (9 U) to the suspension, the mixture was stirred vigorously for 15 h at 40–45 °C. The precipitate was collected by filtration, washed with water, acetone, and chloroform, and then dried under reduced pressure at room temperature to yield the inclusion complex. ^1^H NMR spectra (DMSO-*d*_6_ + D_2_O): amylose–PTHF inclusion complex; δ 1.48–1.55 (br, −C−CH_2_−CH_2_−C− of PTHF), 3.15–3.66 (m, H2−H6 of amylose, overlapping with HOD), 3.30–3.37 (br, O−CH_2_−C−C−CH_2_− of PTHF), 5.11 (br, H1 of amylose); amylose–PCL inclusion complex; δ 1.33–1.36 (br, O=C−C−C−CH_2_ of PCL), 1.55–1.59 (br, O=C−C−CH_2_−C−CH_2_ of PCL), 2.19–2.20 (br, O=C−CH_2_ of PCL), 3.15–3.66 (m, H2−H6 of amylose, overlapping with HOD), 3.58–3.60 (br, C−C*H*_2_−OH of PCL (terminus)), 4.02–4.22 (br, C−CH_2_−O− of PCL), 5.11 (br, H1 of amylose); amylose–PLLA inclusion complex; δ 1.45–1.48 (m, CH_3_ of PLLA), 3.15–3.66 (m, H2−H6 of amylose, overlapping with HOD), 5.11 (br, H1 of amylose), 5.20–5.22 (br, CH of PLLA).

### 3.3. Evaluation of the Stability of Inclusion Complexes 

Mixtures of the aforementioned inclusion complexes (60 mg) with DMSO (4 mL) were stirred for 12 h at desired temperatures (25, 30, 40, 60 °C). Chloroform was then added to the mixtures to obtain precipitates. The precipitates were isolated by filtration and dried under reduced pressure for 3 h at room temperature. The filtrates were concentrated by evaporation and dried under reduced pressure for 5 h at 110 °C. The resulting two fractions were then characterized.

### 3.4. Preparation of ATA Inclusion Supramolecular Polymeric Films

A typical experimental procedure was as follows. Ac_2_O (0.27 mL, 2.86 mmol) was dropped to a mixture of the amylose–polymer inclusion supramolecular polymer (30 mg), DMAP (2 mg, 0.016 mmol), and pyridine (0.5 mL), followed by stirring for 3 days at room temperature. The reaction mixture was diluted with dichloromethane, and extracted with saturated sodium dicarbonate aqueous solution, 1 M hydrochloride aqueous solution, and brine. The organic layer was dried with sodium sulfate, and the filtrate was dried under reduced pressure to yield the ATA inclusion supramolecular polymer. ^1^H NMR spectra (CDCl_3_): ATA–PTHF supramolecular polymer; δ 1.55 (br, –C–CH_2_–CH_2_–C– of PTHF), 1.91–2.15 (m, CH_3_ of Ac), 3.34 (br, OCH_3_), 3.42 (br, O–CH_2_–C–C–CH_2_–O of PTHF), 3.89, 4.20, 4.52, 4.67, 5.24, and 5.33 (amylose); ATA–PLLA supramolecular polymer; δ 1.59 (br, CH_3_ of PLLA), 1.98–2.21 (m, CH_3_ of Ac), 3.95, 4.27, 4.55, 4.72, 5.30, and 5.39 (amylose), 5.16 (q, CH of PLLA). The films were prepared by casting chloroform solutions (33 mg/mL) of the resulting ATA inclusion supramolecular polymers on a glass plate, and dried at room temperature.

### 3.5. Measurements

The ^1^H NMR spectra were recorded using a ECX400 (JEOL, Akishima, Tokyo, Japan), ECA600 (JEOL), and a BioSpin AV-300 spectrometer (Bruker, Yokohama, Kanagawa, Japan). The powder XRD measurements were performed using a X’Pert Pro MPD diffractometer (PANalytical B.V., Almelo, The Netherlands) with Ni-filtered Cu Kα radiation (λ = 0.15418 nm). GPC measurements were conducted using a system consisting of a LC-20AD pump (Shimadzu, Kyoto, Japan), a CTO-10A column oven, an RID-10A refractive index detector, and two TSK gel Super HZM-N (4.6 × 150 mm; TOSOH, Tokyo, Japan) columns. Chloroform was used as the eluent at a flow rate of 0.25 mL/min at 40 °C. Polystyrene samples were used as standards. The DMA were performed using a DMA 2980 Dynamic Mechanical Analyzer (TA Instruments, Tokyo, Japan) at heating rate of 5 °C/min at 1 Hz using a supramolecular polymeric film (5 mm × 20 mm).

## 4. Conclusions

In this paper, we reported the evaluation of the stability of amylose–polymer inclusion complexes under solution state in DMSO. The analytical results of the products indicated that stability in the DMSO solutions increased in accordance with the bulkiness of the guest polymers as follows: PTHF < PCL < PLLA. Furthermore, we achieved preparation of ATA inclusion supramolecular films from the vine-twining supramolecular polymeric products by acetylation of amylose segments. The DMA analysis indicated that the dynamic moduli of the film from ATA inclusion supramolecular polymer depended on the type of included guest polymers.
